# Giant Paratubal Cyst Mimicking Mesenteric Cyst

**DOI:** 10.1155/2022/4909614

**Published:** 2022-10-04

**Authors:** Ebrahim Almahmeed, Abdulaziz Alshaibani, Hamad Alhamad, Abdulmenem Abualsel

**Affiliations:** ^1^Department of General Surgery, King Hamad University Hospital, Bahrain; ^2^Department of Urology, King Hamad University Hospital, Bahrain

## Abstract

Paratubal cysts are adnexal masses located in the broad ligament; whenever the size of the cyst is >20 cm, it is considered a giant cyst and can present with complications including ovarian torsion and perforation. Diagnosis can be made clinically with the help of radiological investigations, although the gold standard diagnostic tool is diagnostic laparoscopy. Managing this condition relies mainly on surgical intervention (open or laparoscopic). Giant paratubal cysts carry challenges in their surgical excision as they carry a higher risk of spillage. We present a case of giant paratubal cyst in a 26-year-old Bahraini female who presented with abdominal distension and pain for 3 years.

## 1. Introduction

Paratubal cysts are adnexal masses that originate from paramesonephric, mesonephric, or mesothelial origins [1]; they constitute 5-20% of all adnexal masses [2]. Most of them are asymptomatic and are small in size [3]; they can present with abdominal pain (secondary to torsion, rupture) or intra-abdominal pressure symptoms. Paratubal cysts can be mistaken for ovarian masses, and it is difficult to differentiate between them clinically and radiologically [3]. Therefore, laparoscopic surgical excision is the preferred approach for paratubal cysts [4].

## 2. Case Presentation

A 26-year-old woman who has no known case of any chronic medical illnesses presented to the general surgery clinic with the complaint of progressive abdominal distension for 3 years. Additionally, the patient noticed abdominal distension with on and off vague generalized abdominal pain. The patient did not seek prior medical advice for her abdominal distension. There were no other associated complaints and no history of trauma.

**Figure 1:**
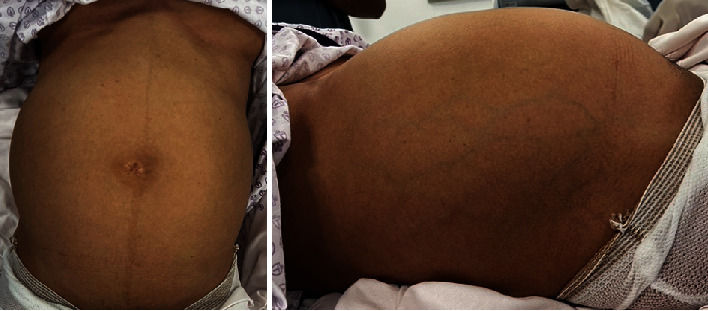
Abdominal inspection showing diffuse abdominal distension.

The patient is single and nulliparous with no history of sexual intercourse, no history of hormonal supplementation, and no past surgical history. Upon abdominal examination, distension involving the lower abdomen was present and reached the epigastric area. There was no tenderness or skin changes that could be appreciated (Figure 1). Rectal examination was unremarkable, vaginal examination was deferred as the patient was single with intact hymen.

**Figure 2:**
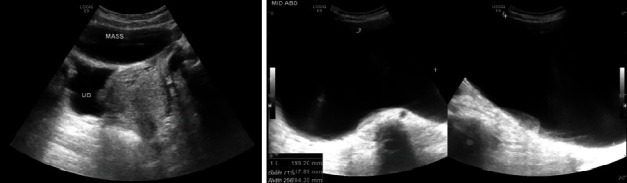
Abdominal and pelvic ultrasonography showed a huge cystic lesion with clear fluid contents.

The patient was investigated initially with laboratory tests, abdominal and pelvic ultrasonography, and pelvic magnetic resonance imaging (MRI). Blood tests were all within normal range (complete blood count, renal function tests, liver function tests, and electrolytes), in addition to negative serum tumor markers (AFP 2 ng/mL, CA125 13.4 U/mL, LDH 164.6 U/L, total hCG 0, and CEA 0). The abdominal and pelvic ultrasonography (Figure 2) showed a huge cystic lesion with clear fluid contents occupying the abdominal and pelvic cavity and displacing the abdominal organs and bowel loops with no soft tissue component.

**Figure 3:**
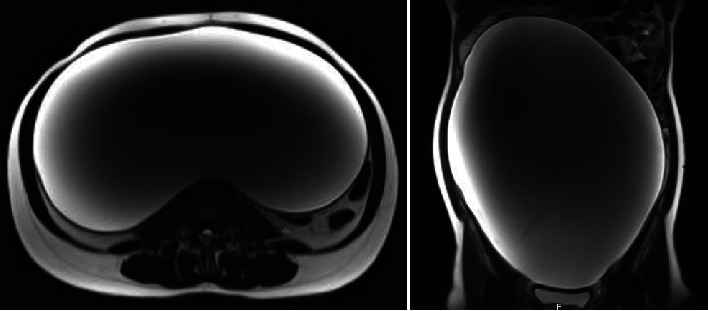
Pelvic magnetic resonance imaging showing a large well defined pelviabdominal cystic lesion.

MRI of the pelvis (Figure 3) showed a large well-defined pelviabdominal cystic lesion with homogenous content. The lesion measured about 16 × 26 × 34 cm (in AP × ML × CC dimensions) and extended upward abutting and displacing the transverse colon. The anatomical location of the lesion reached the subhepatic region superiorly; anteriorly, it extended abutting the anterior abdominal wall, laterally reached both lateral abdominal wall, and inferiorly to the lower pelvis. It showed marked mass effect on the adjacent structures; however, it was seen separable from the surrounding bowel loops. Radiological signs of malignant transformations were not present but showed relatively thickened walls with no enhanced solid component.

**Figure 4:**
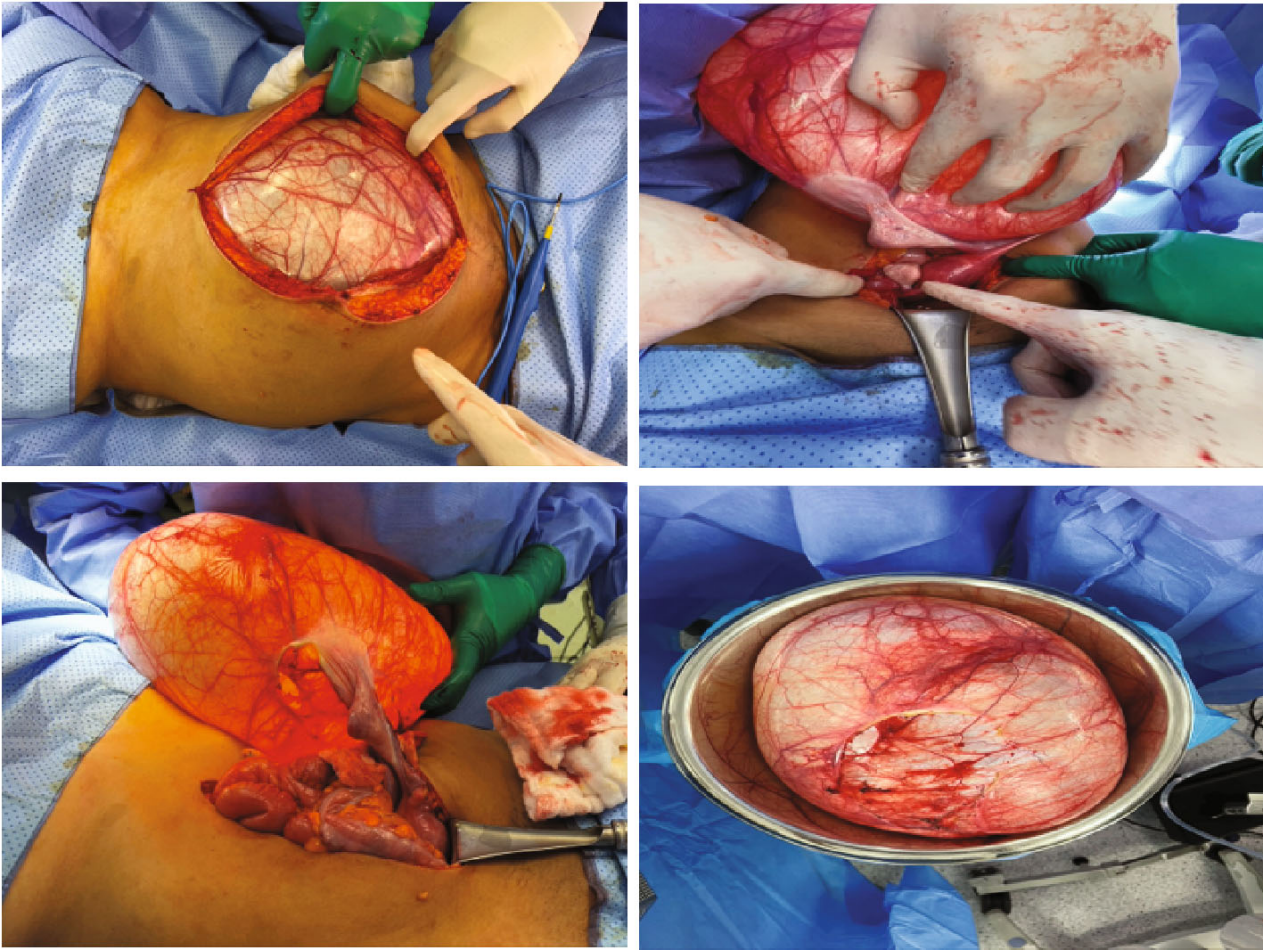
Intraoperative findings of left huge paratubal cystic mass.

The decision to start with laparotomy instead of diagnostic laparoscopy was made due to the large size of the mass and the uncertainty of the pathology of the tumor (malignant or benign); the patient underwent elective exploratory laparotomy with excision of the mass; intraoperatively, the mass was found to be left paratubal (fallopian tube) and measuring 40 × 20 cm with 7 liters of clear content, with crossing blood vessels over the cyst (Figure 4).

The patient was discharged second day postoperatively in a stable condition and with no complaints; she was followed up in the outpatient general surgery clinic regularly until full healing of the wound; the patient had no complaints during her visits.

In view of the patient's history, physical examination, and radiological investigations, the differential diagnoses were mesenteric cyst and ovarian cyst. However, the histopathology sections revealed wall of cyst lined by a single layer of cuboidal cells with focal area showing ciliated cells. No papillae or atypia was seen. One of the sections revealed part of the fallopian tube; thus, the final diagnosis was benign paratubal cyst.

Postoperatively, the patient was discharged home on the 3rd day in a stable condition; follow-ups in the clinic revealed resolution of the symptoms.

## 3. Discussion

Paratubal cysts (PTCs) are fluid-filled masses that occur in the mesosalpinx within the broad ligament [5]. Overall, most of these cysts are found incidentally in up to 92% of adult women in hysterectomy specimens or found on ultrasound, and the peak age of diagnosis is between the third and fourth decades [6, 7]. They usually range from 1 to 8 cm^5^, and only a few cases of giant paratubal cysts (>20 cm) have been described [8]. The largest paraovarian cyst has been reported in the literature in 2006 by Letourneur et al. which was 36 × 9 × 25 cm [9]. In our case, the patient was in her 3rd decade, with a cyst detected radiologically as 16 × 26 × 34 cm and upon physical and pathological inspection as 40 × 20 cm.

PTCs originate from the paramesonephric (Mullerian) embryological remnants in up to 76% of the cases, whereas the remaining types are mesothelial or rarely mesonephric (Wolffian) in origin [10]. Cysts that originate from paramesonephric remnants are lined with ciliated columnar or cuboidal epithelium, whereas mesonephric remnant cysts are lined with cuboidal or flattened epithelium [10]. It is thought that the paramesonephric or mesonephric duct remnants increase in size with hormonal activity giving rise to PTCs [10].

Most of the PTCs are small and asymptomatic and rarely require treatment [11]. The larger the cysts get, the more symptomatic they become, as they can cause pressure symptoms on the surrounding organs including the bladder, uterus, or bowel, causing pelvic and abdominal pain [11]. Complications are also proportionally associated with the size of the cysts, as they can lead to hemorrhage, rupture, and torsion, although complications are rare [12]. In our patient, the histopathology was cuboidal cells with focal areas of ciliated cells.

Physical examination might be unremarkable, but as the PTCs grow in size, abdominal and adnexal tenderness can be elicited on pelvic examination, in addition to palpation of the cyst. In case of complicated PTCs (torsion, rupture, and hemorrhage), examination may reveal peritoneal signs [13]. Laboratory values are usually unspecific and of no diagnostic value. In our patient, a palpable cyst that was occupying most of the abdomen due to the size of the cyst with normal laboratory tests was noted.

PTCs can be detected preoperatively with radiological investigations, with ultrasonography and magnetic resonance imaging (MRI) being more useful than CT scan [12]. PTCs are thin walled, unilocular, anechoic, or hypoechoic on imaging [14]. Although ultrasound can guide the surgeon about the location and the characteristics of the mass, a study that was performed at the University of Iowa indicated that even transvaginal ultrasound misdiagnosed paratubal and paraovarian cysts 93% of the time [15]. The differential diagnosis of homogeneous, unilocular cysts includes ovarian cysts, paratubal and para ovarian cysts, and mesenteric cyst. Further surgical exploration is required to confirm the origin and the pathology of the cyst [16]. The intraoperative finding of blood vessels crossing over the cyst is a pathognomonic sign of paratubal cyst to be differentiated from ovarian cyst [17]. In our case, the patient was investigated with ultrasound and MRI of the abdomen and pelvis, both of which showed a cystic lesion with homogenous clear content, with a differential diagnosis between mesenteric and ovarian cysts; intraoperatively, the cyst was found to be paraovarian, and its wall showed crossing of blood vessels which is pathognomonic for paratubal cyst.

The treatment approach for symptomatic PTCs is surgical excision via laparotomy or laparoscopic approach; in case of symptomatic PTCs, excision has not been recommended by some authors [18]. Laparotomy has been used for giant paratubal cysts excision for multiple reasons including the difficulties with achieving pneumoperitoneum and inserting the trochars and the risk of rupture with no definitive diagnosis. Aspiration of the cyst has been recommended to prevent any intraperitoneal spillage [19].

Whenever the size of the cyst allows laparoscopic excision, it is preferred as it has the advantages of smaller incisions, shorter hospital stay, and faster patient's recovery [20]. Absolute contraindication to laparoscopic approach is related to contraindications to establish pneumoperitoneum such as hemodynamic instability or cardiopulmonary impairment [21]. Consequently, a laparotomy approach was used in our case due to the size of the mass and the risk of rupture with no clear preoperative diagnosis. Part of the fallopian tube attached to the cyst was excised to assured complete excision and prevent any further recurrence.

## 4. Conclusion

Paratubal cysts are masses that originate in the broad ligament and are common in the 3rd and 4th decades. Giant PTCs (more than 20 cm) are rare and difficult to diagnose and differentiate from other abdominal and pelvic pathologies. Complications of this diagnosis can be torsion, hemorrhage, and perforation. Prompt diagnosis is required and can be aided with imaging studies that include ultrasonography and MRI. Surgical excision is the mainstay of management of PTCs and can be performed with laparotomy or laparoscopy if feasible.
